# Disposable Voltammetric Immunosensor for D-Dimer Detection as Early Biomarker of Thromboembolic Disease and of COVID-19 Prognosis

**DOI:** 10.3390/bios13010043

**Published:** 2022-12-28

**Authors:** Cristina Tortolini, Valeria Gigli, Antonio Angeloni, Luciano Galantini, Federico Tasca, Riccarda Antiochia

**Affiliations:** 1 Department of Experimental Medicine, Sapienza University of Rome, V.le Regina Elena 324, 00166 Rome, Italy; 2 Department of Chemistry, Sapienza University of Rome, P.le Aldo Moro 5, 00185 Rome, Italy; 3 Departamento de Química de los Materiales, Facultad de Química y Biología, Universidad de Santiago de Chile, Casilla 40, Correo 33, Sucursal Matucana, Santiago 9170022, Chile; 4 Department of Chemistry and Drug Technologies, Sapienza University of Rome, P.le Aldo Moro 5, 00185 Rome, Italy

**Keywords:** D-dimer, chitosan nanoparticles, voltammetric immunosensor, MWCNTs-screen printed electrode, COVID-19, human plasma samples

## Abstract

In this work, we report on the development of a simple electrochemical immunosensor for the detection of D-dimer protein in human plasma samples. The immunosensor is built by a simple drop-casting procedure of chitosan nanoparticles (CSNPs) as biocompatible support, Protein A (PrA), to facilitate the proper orientation of the antibody sites to epitopes as a capture biomolecule, and the D-dimer antibody onto a carboxyl functionalized multi-walled carbon nanotubes screen printed electrode (MWCNTs-SPE). The CSNPs have been morphologically characterized by Scanning Electron Microscopy (SEM) and Dynamic Light Scattering (DLS) techniques. Successively, the electrochemical properties of the screen-printed working electrode after each modification step have been characterized by differential pulse voltammetry (DPV) and electrochemical impedance spectroscopy (EIS). The resulting MWCNTs-CSNPs-PrA-D-dimer Ab immunosensor displays an optimal and promising platform for antibody immobilization and specific D-dimer detection. DPV has been used to investigate the antigen/antibody interaction at different D-dimer concentrations. The proposed voltammetric immunosensor allowed a linear range from 2 to 500 μg L^−1^ with a LOD of 0.6 μg L^−1^ and a sensitivity of 1.3 μA L μg^−1^ cm^−2^. Good stability and a fast response time (5 s) have been reported. Lastly, the performance of the voltammetric immunosensor has been tested in human plasma samples, showing satisfactory results, thus attesting to the promising feasibility of the proposed platform for detecting D-dimer in physiological samples.

## 1. Introduction

D-dimer is a fibrin degradation product (FDP) present in the blood after a blood clot breakdown. After a blood vessel injury, the blood clotting system can limit excessive blood loss. The body produces several proteins that come together at the site of the injury to create a blood clot. After the wound has healed, a different protein called plasminogen, after converting to the active plasmin, allows fibrinolysis to occur, breaking down the clot into fibrin degradation products. One of them is called fragment D, and dimers of this fragment joined by a cross-linker are known as D-dimer [1].

Since the 1970s, D-dimer has been used as a biomarker to activate hemostasis and fibrinolysis [1]. A strong correlation was found between its high levels in the blood and the occurrence of deep vein thrombosis (DVT), venous thromboembolism, disseminated intravascular coagulation (DIC), stroke, and thrombosis in cancer [2,3,4,5,6,7,8,9,10]. D-dimer elevated levels are also correlated with increased mortality risk in patients with cancer in the lung [5,6], breast [7], lower gastrointestinal tract [8,9], pancreas, stomach, kidney, prostate, and brain [2]. Moreover, D-dimer levels are nearly always increased in the presence of acute pulmonary embolism (PE) [11,12]. PE is a disease that presents non-specific symptoms, such as difficulty in breathing, chest pain, and/or palpitations. Therefore, an initial diagnostic test for patients with suspected acute PE is extremely important. It was found that a D-dimer value lower than 0.5 μg mL^−1^ can rule out PE, whereas patients with D-dimer levels over 1.96 μg mL^−1^ (4 times the normal level) have a significant risk for PE [13]. 

Recently, D-dimer was also found to be an important biomarker for assessing COVID-19 prognosis [14]. Values in normal patients are in the range of 0–0.243 μg mL^−1^, whereas in infected COVID-19 patients they are generally higher than 0.5 μg mL^−1^ [15]. The elevation in D-dimer values at the time of admission to the hospital was investigated as a possible prognostic indicator of severity and/or mortality in COVID-19 patients [16]. Increased D-dimer levels are associated with thromboembolic complications, but they might also be a direct consequence of the acute lung injury seen in COVID-19 pneumonia [17]. The cut-off value used for D-dimer showed significant variation between published studies, meaning that the optimal cut-off for D-dimer still needs to be definitively established [18,19]. However, the in-hospital mortality was significantly higher in patients with D-dimer ≥ 2.0 μg mL^−^^1^ on admission [20]. Therefore, a value of 2.0 μg mL^−^^1^ was considered the optimal probability cut-off for judging an outcome of death [19].

Of course, due to the rapid spread of COVID-19, accurate and widely available prognostic biomarkers can be very useful tools in COVID-19 management. Among routine tests, D-dimer was one of the best early biomarkers.

Nevertheless, the clinical use of D-dimer is still mediocre, probably because of the lack of reliable, simple, low-cost point-of-care (POC) assays for D-dimer quantification [9]. 

D-dimer assays were initially based on enzyme-linked immunosorbent assay (ELISA) technology, which became the gold standard for D-dimer testing [21]. Successively, this method was replaced by more rapid and cost-effective techniques based on immunoturbidimetry, latex agglutination, chemiluminescence, or immunofluorescence, where D-dimer is reported as Fibrinogen Equivalent Units (μg mL^−1^) [22,23,24,25]. Although these assays are highly sensitive, they still require a prolonged response time of more than 40 min. In this context, biosensors are versatile tools for biomarker detection, which combine high sensitivity and selectivity with rapid responses within a few minutes. Moreover, they require low-cost instrumentation, can be easily miniaturized for point-of-care (POC) use, and are well suited for direct electrical readout [26]. The incorporation of nanoscale materials into the biosensing platform allowed for greatly enhancing the biosensor sensitivity, thanks to the possibility of immobilizing an enhanced quantity of bioreceptors and even acting themselves as transduction elements [27]. Among such nanomaterials, gold nanoparticles [28], carbon nanotubes [29,30], and graphene [31] have been mostly utilized in biosensing development for D-dimer detection.

The present work aimed to develop a disposable voltammetric immunosensor for D-dimer detection based on the deposition of chitosan nanoparticles onto the surface of disposable MWCNTs screen-printed electrodes. Detection was due to the irreversible binding of the D-dimer antigen to its corresponding antibody. No label was added for the detection, thus resulting in a simplified, low cost and disposable electrochemical platform. 

The electrochemical performances of the voltammetric immunosensor were initially investigated and optimized in phosphate buffer, also utilizing a portable potentiostat directly connected to a smartphone for POC signal reading.

The proposed immunosensor was successively tested in real blood plasma samples of healthy and COVID-19-infected patients, attested by positive RT-PCR results. The response of the immunosensor was compared with a conventional immunoturbidimetric method, showing excellent agreement.

## 2. Experimental

### 2.1. Reagents

The anti-D-dimer antibody (D-dimer Ab) and D-dimer antigen (D-dimer Ag) were furnished by Abcam (Cambridge, UK). Chitosan (CS, low molecular weight deacetylated chitin, Poly(D-glucosamine), potassium chloride (KCl), sodium tripolyphosphate (TPP, 85%), potassium ferricyanide (III) (K_3_[Fe(CN)_6_], 99.0%), potassium ferrocyanide (II) (K_4_[Fe(CN)_6_], 98.5–102.0%), Protein A (Pr A), Bovine Serum Albumin (BSA), sodium phosphate monobasic (NaH_2_PO_4_, ≥99%), sodium phosphate dibasic (Na_2_HPO_4,_, ≥99%), IgG, fibrinogen (Fb), and potassium chloride (KCl, 99–100.5%) were purchased from Sigma Aldrich (Bucks, Germany). Each solution was made in phosphate buffer 0.1 M, KCl 0.1 M, and pH 7.4 (PBS). Throughout the investigations, high-purity deionized water from Millipore (Molsheim, France) was utilized (resistance: 18.2 MΩ cm at 25 °C; TOC < 10 µg L^−1^). All chemicals used were analytical grade and were used as received without any further purification.

For the smartphone-based sensing device measures, a Sensit/SMART portable potentiostat (PalmSens, Houten, The Netherlands) was employed and directly connected to a smartphone for POC signal reading.

### 2.2. Electrochemical Measurements and Apparatus

All electrochemical measurements were carried out in a 10-mL thermostated glass cell (model 6.1415.150, Metrohm, Herisau, Switzerland). A carboxyl functionalized multi-walled screen-printed electrode (MWCNTs-SPE, CNT110 Metrohm, Herisau, Switzerland, Work: MWCNTs-COOH/carbon; Aux: carbon; Ref: silver, diameter 4 mm) was used. The measurements were performed with an Autolab PGSTAT204 potentiostat operated by Nova v 2.1 (Metrohm, Herisau, Switzerland). 

Electrochemical impedance spectroscopy (EIS) data were carried out in the frequency range 0.1–10^5^ Hz, using an AC signal of 10 mV amplitude and under open-circuit potential (OCP) conditions.

Cyclic voltammetry (CV), Differential Pulse Voltammetry (DPV), and Electrochemical Impedance Spectroscopy (EIS) measurements were performed using 10 mL of a solution containing a mixture of 5 mM Fe(CN)_6_^3−^/Fe(CN)_6_^4−^, 0.1 M KCl in distilled water as an electrochemical redox probe (Zobell’s solution). Zobell’s solution was used in CV experiments for determining the electroactive area (A_e_) using the Randles–Ševcik equation and for determining the roughness factor (ϱ = A_e_/A_geom_).

The particle size distribution of the CSNPs and their zeta potential were determined using Dynamic Light Scattering (DLS) and electrophoretic mobility measurements based on the laser Doppler electrophoresis technique, respectively, using a Zetasizer Nano ZS90 (Malvern Instruments Ltd., Malvern, UK). The instrument was equipped with a 5 mW HeNe laser (λ = 632.8 nm) and a digital logarithmic correlator; the measurements were performed at 25.0 ± 0.1 °C. The intensity autocorrelation function was analyzed by the non-negative least square (NNLS) algorithm to obtain the distribution of the particle diameters. In the laser Doppler electrophoresis technique, the Doppler shift in the Zetasizer Nano series was analyzed by phase analysis light scattering. Zeta potential values were inferred from the electrophoretic mobility data under the Smoluchowsky approximation. Averages over 3 consecutive measurements were reported for each sample.

The morphological features of unmodified and modified electrodes were studied by scanning electron microscopy (SEM) and energy-dispersive X-ray spectroscopy (EDX) measurements using a High-Resolution Field Emission Scanning Electron Microscopy (HR FESEM, Zeiss Auriga Microscopy, Jena, Germany).

A freeze-dried chitosan nanoparticles FTIR spectrum was acquired in Attenuated total reflectance mode (ATR–FTIR) using a Thermo Nicolet 6700 FT-IR Spectrometer equipped with a Specac Golden Gate diamond single reflection accessory. Spectra were acquired in the range 4000–650 cm^−1^ by co-adding 100 scans with a resolution of 4 cm^−1^. 

The human plasma samples were tested by immunoturbidimetry using a Siemens BCS XP System (Siemens Healthcare s.r.l., Milan, Italy) and a kit INNOVANCE Siemens for D-dimer Assay.

### 2.3. Preparation of Chitosan Layer

Chitosan layer (CS layer, 10 μL, 10 mg mL^−1^ in 1% (*w*/*v*) acetic acid) was drop-casted on the MWCNTs-SPE surface and incubated for about 30 min at room temperature (RT). Then the SPE was rinsed with distilled water.

### 2.4. Synthesis of Chitosan Nanoparticles 

The chitosan nanoparticles (CSNPs) were synthesized using an ionic gelation process, as described in our previous work [32].

### 2.5. Immunosensor Fabrication Steps

**Scheme 1:**
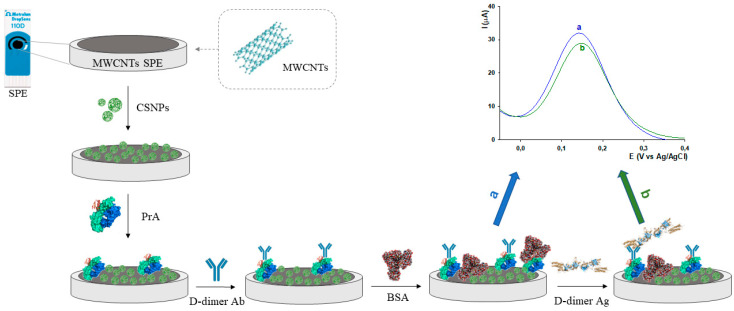
Schematic representation of the D-dimer electrochemical platform fabrication steps and immunosensor sensing mechanism.

The immunosensor was prepared according to the following steps: (i) 10 μL of CSNPs were drop-casted on the working area of the MWCNTs-SPE and let dry at RT (30 min); (ii) 10 μL of PrA (0.5 mg mL^−1^) were drop-casted on the working area followed by 45 min of drying at RT; (iii) 10 μL of D-dimer Ab (0.1 μg mL^−1^) were dropped on the working area (20 min); (iv) the sensor was immersed in a BSA solution (10 μL, 0.25% *w*/*v*) for 15 min, as a blocking agent, to avoid non-specific interactions. The surface electrode was washed with a buffer solution at the end of each fabrication step and stored at 4 °C before use. The overall modification steps of the working electrode for the development of the D-dimer immunosensor are reported in Scheme 1. All bio-compounds used are classified as non-hazardous materials.

### 2.6. Preparation of Human Plasma Samples 

Five human plasma samples, obtained from Policlinico Hospital of Rome, were suitably diluted with 1:2, 1:3, and 1:4 dilutions with PBS pH 7.4 before all measurements. 

## 3. Results and Discussion

### 3.1. SEM Characterization of CSNPs

**Figure 1:**
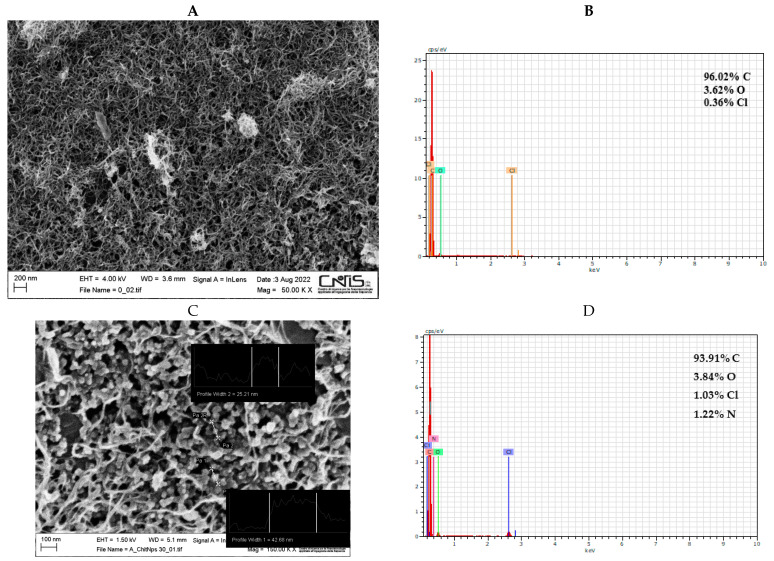

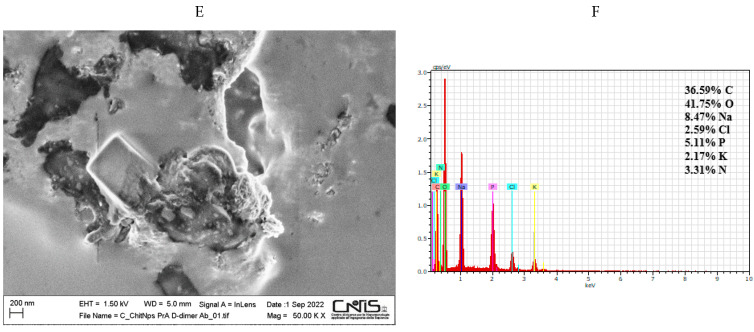
SEM images and EDX spectra for MWCNTs (**A**,**B**), MWCNTs-CSNPs (**C**,**D**), and MWCNTs-CSNPs-PrA-D-dimer Ab (**E**,**F**) SPEs. Experimental conditions: magnifications 50,000× (**A**,**E**) and 150,000× (**C**); voltage 1.50 kV.

Field emission scanning electron microscopy (SEM) coupled with energy-dispersive X-ray technique (EDX) was used to examine the morphological structure of bare and modified electrodes. A uniform layer of carbon nanotubes was visible on the surface of the bare electrode (Figure 1A). The EDX spectra confirmed the presence of carbon and oxygen elements with an atomic percentage, calculated from the quantification of the peaks, of about 96% and 4%, respectively. The presence of a low amount of Cl element (<1%) is probably due to possible impurities of the commercial screen-printed electrode (Figure 1B). The SEM image of MWCNTs-CSNPs-SPE clearly shows aggregates of nanoparticles of spherical shape with diameters ranging from 25 to 50 nm (Figure 1C). The corresponding EDX spectra (Figure 1D) show the presence of an N atom, confirming the immobilization of the chitosan molecule on the sensor surface. Finally, the FESEM image of the MWCNTs-CSNPs-PrA-D-dimer Ab-SPE-based platform presents a clearly visible macro-structured surface, confirming the immobilization of large biomolecules, such as the PrA and the antibody, which properly cover the electrode surface (Figure 1E). Additionally, the EDX mapping of the latter platform demonstrates the presence of multiple heteroatoms (Na, P, and K) and an enhanced amount of N element, indicating that both PrA and the antibody were successfully immobilized on the nanostructured electrode surface (Figure 1F).

### 3.2. DLS and FTIR Characterization of CSNPs

**Figure 2:**
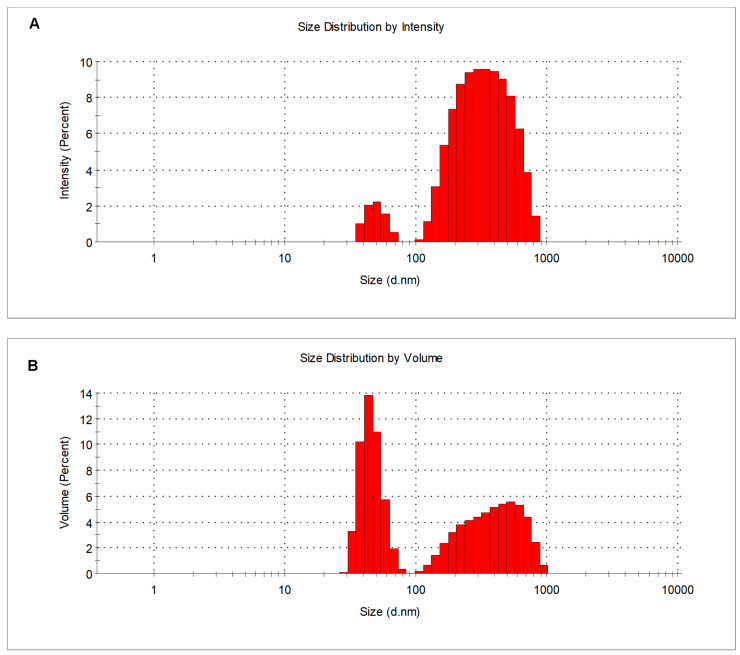
DLS size distribution analysis of 1 mg mL^−1^ CSNPs solution by (**A**) intensity and (**B**) volume.

The synthesized CSNPs were dispersed in water and characterized by DLS. Intensity distributions of the particle diameters extracted by this technique showed the presence of two populations around 50 and 360 nm (Figure 2A). This bimodal distribution suggests that small primary particles are formed, which comprise the population with smaller diameters. These primary particles are partially assembled into clusters forming the population at larger sizes. The volume-weighted distributions show that the fraction of clusters is comparable with that of the primary particles (Figure 2B). A slightly negative zeta potential value of 2–3 mV was measured for dispersions. These low values suggest insufficient stability of the particle dispersions and justify their observed aggregation into clusters.

SEM experiments confirm the presence of primary particles with sizes around 25–50 nm. As SEM is performed on dried samples, the sizes are slightly smaller than those shown by DLS, probably due to some dehydration-induced shrinkage of the nanoparticles. The nanoparticles can be recognized upon a bed of assembled material, probably formed because of a massive aggregation of nanoparticles again promoted by drying.

FTIR studies of CSNPs implemented the investigation of their chemical structure (Figure S1). The FTIR spectrum denotes the major IR absorption bands typical of chitosan nanostructure attributed to O-H stretching (3430 cm^−1^), -N-H3+ stretching (2700–2300 cm^−1^), N-H bend (1630 cm^−1^), and C-O stretching (1100 cm^−1^).

### 3.3. Electrochemical Characterization of CSNPs

Preliminary surface characterization of bare and modified MWCNTs-SPEs was performed using CV and EIS techniques, which provide helpful information on the electrochemical behavior of the MWCNTs-SPE before and after the functionalization with CSNPs. The MWCNTs-SPEs were also functionalized with a single layer of unstructured chitosan (CS layer) as a comparison.

#### 3.3.1. CV Characterization

**Figure 3:**
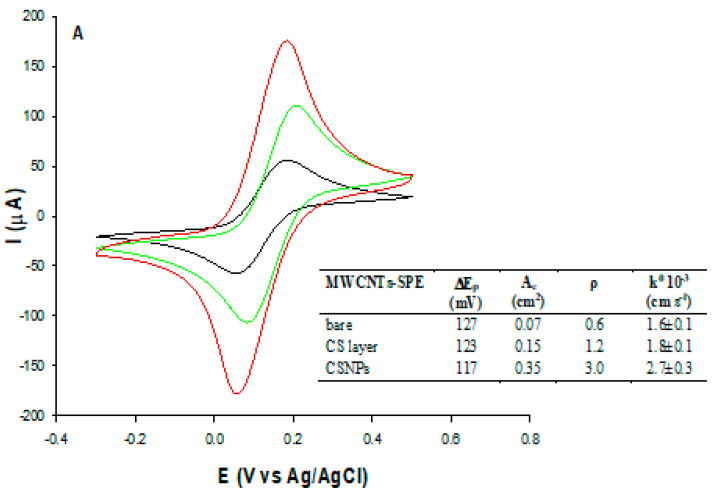

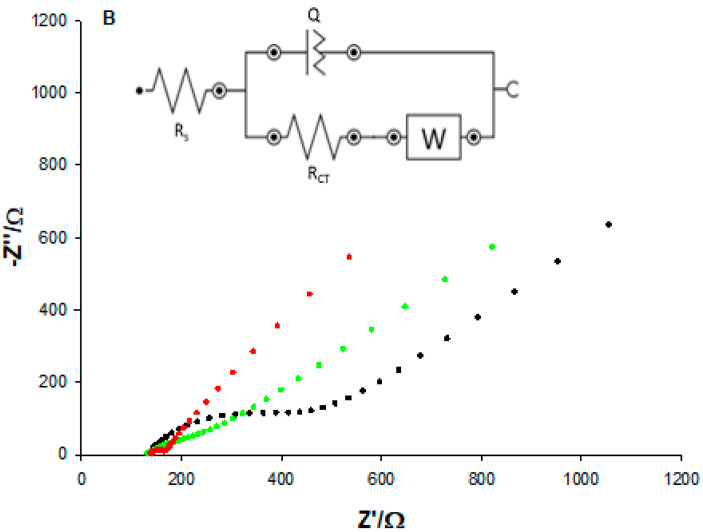
(**A**) CVs and (**B**) Nyquist plots of MWCNTs (black), MWCNTs-CS layer (green), and MWCNT-CSNPs (red) SPEs, measured in Zobell’s solution. Inset: simple Randles circuit.

Figure 3 shows the cyclic voltammograms obtained before (black curve) and after the modification of MWCNTs-SPE with unstructured (MWCNTs-CS layer, green curve) and nanostructured chitosan (MWCNTs-CSNPs, red curve). CV experiments are carried out in Zobell’s solution by cycling from −0.3 to 0.5 V vs. Ag/AgCl, at a scan rate of 0.05 V s^−1^. All CVs show a couple of quasi-reversible redox peaks, with peak-to-peak separations (ΔE_p_) slightly decreasing and passing from the unmodified to the CS layer and the CSNPs modified electrodes, respectively (see inset Figure 3A). An opposite trend was obtained with the peak current values, resulting in the lowest peak current with the bare electrode (black curve), the intermediate value with the CS layer modified electrode (green curve), thanks to the conductivity of the CS layer, and the highest value with CSNPs modified electrode (red curve), because of the improved conductivity of chitosan nanoparticles compared to pristine chitosan. The electroactive area (A_e_) and roughness factor (ρ) have been successively calculated from the CV curves and reported in the table of Figure 3A inset. The A_e_ has been calculated by plotting the peak current vs. square root of the scan rate (*v*^1/2^) and inserting the slope value obtained into the following Randles-Sevcik equation [33]:

I_p_ = 2.686 × 10^5^ n^3/2^ A_e_ D_0_ ^1/2^ C_0_ *v* ^1/2^ (1)

where I_p_ is the voltammetric peak current (A), n is the number of electrons, A_e_ is the electroactive area (cm^2^), D_0_ is the diffusion coefficient (7.6 × 10^−6^ cm^2^ s^−1^ for ferricyanide), C_0_ is the concentration (mol cm^−3^), and *v* is the scan rate (V s^−1^), while the roughness factor (ρ) was evaluated as electroactive/geometric area ratio. The A_e_ values of MWCNTs-CS layer-SPE and MWCNTs-CSNPs-SPE were about 2 and 5 times higher than the bare electrode, respectively. The coverage of the electrode surface with the conductive chitosan film resulted in an obvious increase in the A_e_ values, which became significantly higher when nanostructured chitosan was used. The first effect can be ascribed to the well-known natural micrometric porous structure of chitosan, which leads to an increase of the electrode surface area [34], whereas the latter can presumably be attributed to the upgraded properties of chitosan by making its nanoparticles, thanks to the synergic effect of nanosize and large surface area/volume ratio [35]. Table 1 also shows the heterogeneous electron transfer rate constants (k^0^, cm s^−1^) for the MWCNTs-SPE before and after the modification with the CS layer and CSNPs, calculated using the Lavagnini et al. method, which merges the Klingler–Kochi and Nicholson and Shain methods for irreversible and reversible systems, respectively [36]. The MWCNTs-CSNPs-SPE electrode showed the best performance also in terms of the highest k^0^ value (k^0^ = 2.7 ± 0.3 10^−3^ cm s^−1^), confirming that the nanostructuration promotes a faster electron transfer (inset Figure 3A).

#### 3.3.2. EIS Characterization

Further characterization of the bare and the two modified MWCNTs-SPEs was performed using the EIS technique. The EIS consists of a semicircle portion occurring at a higher frequency range corresponding to the electron-transfer-limited process and a linear part at lower frequencies representing the diffusion-limited process. The semicircle of the Nyquist plot represents the charge transfer resistance (R_CT_) of the system, measured as semicircle diameter. Figure 3B shows the typical Nyquist plots relative to the impedance spectra for MWCNTs bare (black curve), MWCNTs-CS layer (green curve), and MWCNTs-CSNPs (red curve) SPEs in Zobell’s solution. The data obtained were fitted with the Randles equivalent circuit [R(Q[RW])], which includes the electrolyte solution resistance (R_s_), the charge transfer resistance (R_CT_), the constant phase element (Q), and the Warburg element (W) (inset Figure 3B). The Randles parameters are reported in the first three rows of Table 1. The bare SPE shows the largest semicircle (R_CT_ = 324 Ω), corresponding to the lowest electron transfer rate between the redox probe and the electrochemical double layer. After the functionalization with the CS layer, the R_CT_ value slightly decreased (229 Ω) thanks to the better conductivity of pristine chitosan. A further marked decrease was obtained when the electrode surface was modified with CSNPs (35 Ω), thanks to the formation of electrostatic interactions between CSNPs and the -COOH functional groups of MWCNTs, showing the smallest semicircle and proving that the CSNPs increase the conductivity of the electrode/electrolyte interface, due to their better electroconductive properties, compared to pristine chitosan. As for the Q value, which is the capacitance of the MWCNTs-SPE/electrolyte interface, an increase was observed after the modifications of the electrode surface (about 4 and 26 times for the CS layer and CSNPs, respectively), reflecting the increased roughness values (inset Figure 3A) of the platforms, definitely higher after the nano-modification step.

Warburg impedance (W) tends to increase as the film (i.e., MWCNTs) is functionalized. In fact, surface adsorption of polymers (chitosan) and/or proteins (PrA and BSA) hinders electrolyte diffusion. It is worth noticing that W rises as the molecular weight of the adsorbed molecule increases. Furthermore, the combination of more than one macromolecule on the electrode surface cause W to increase (and the slope to deviate from 45°) by limiting access to active sites. Similarly, electrolyte penetration through nanostructured chitosan (CSNPs) is disfavored (compared to the CS layer), leading to a higher Warburg impedance modulus. In addition, MWCNT-CSNPs clearly show a high-frequency RC behavior probably due to interfacial polarization (i.e., Maxwell–Wagner) stemming from chitosan nanoparticles embedded in the electrode. Indeed, Y0 turned out to be the largest for CSNPs, reflecting the interfacial contribution to capacitance (CPE). Conversely, bare (uniform) electrodes (only MWCNTs) showed no interfacial polarization effect in accordance with Y0 low values. Moreover, CSNPs’ nanostructured nature allowed for a fast charge transfer reaction at the electrode/electrolyte interface resulting in the lowest R_CT_ value. 

### 3.4. Electrochemical Characterization of the D-Dimer Immunosensors Platform 

The electrochemical MWCNTs-SPE electrode behavior after each surface modification step performed for the construction of the D-dimer immunosensor was studied by differential pulse voltammetry (DPV) and electrochemical impedance spectroscopy (EIS) techniques in Zobell’s solution.

#### 3.4.1. DPV Characterization

**Figure 4:**
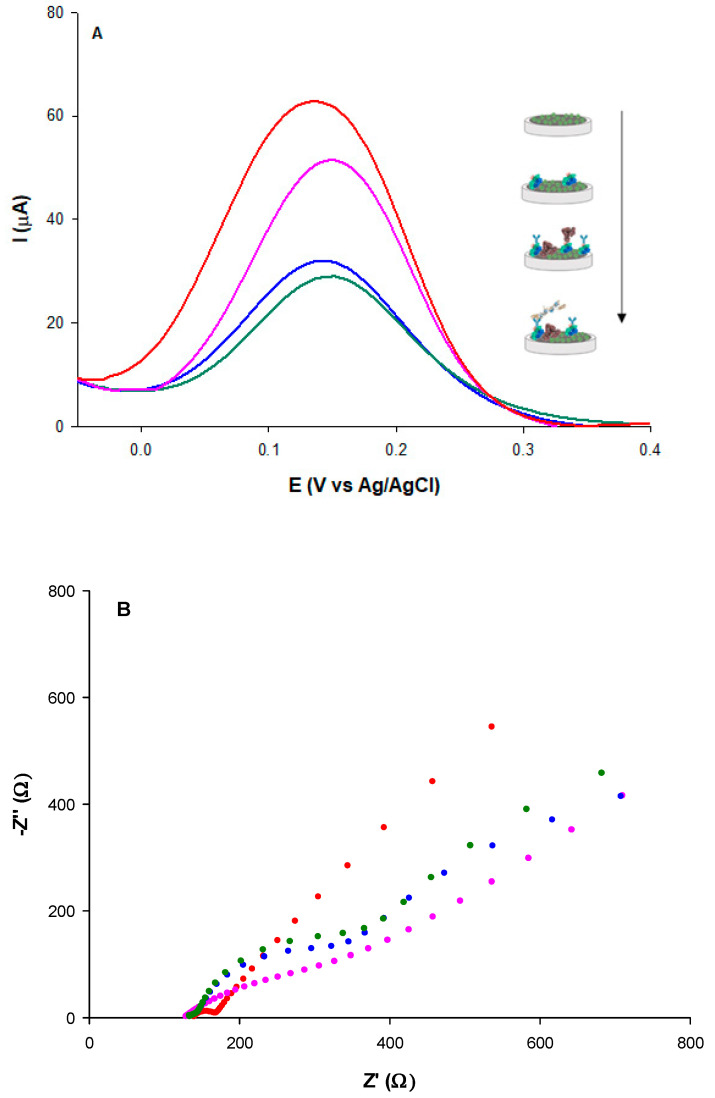
(**A**) DPVs and (**B**) Nyquist plots of: MWCNTs-CSNPs (red), MWCNTs-CSNPs-PrA (pink), MWCNTs-CSNPs-PrA-Ab-BSA (blue), MWCNTs-CSNPs-PrA-Ab-BSA-Ag (dark green) SPEs, measured in Zobell’s solution; D-dimer antigen = 100 μg L^−1^; D-dimer antibody = 0.1 μg mL^−1^.

Figure 4A shows the DPV curves for the assembly of the D-dimer immunosensor. Compared to the unmodified MWCNTs-CSNPs platform (red curve), a progressive decrease of the peak current is registered in correspondence to the following steps: (i) attachment of protein A (pink curve); (ii) anchoring of D-dimer antibody/BSA (blue curve); (iii) formation of D-dimer Ab-Ag complex (dark green curve). These results indicate that the electron transfer through the electrode surface is hampered by the insulating nature of PrA, antibody, BSA, and antigen molecules. With the MWCNTs-CSNPs-Ab-BSA platform in the absence of PrA, a slightly larger decrease of the DPV current was observed, attesting to a larger amount of antibodies immobilized onto the modified electrode surface, probably because of the absence of the steric effect of PrA (Figure S2, black curve). After the binding of the D-dimer antigen, a smaller current variation was observed compared to the corresponding curve obtained in the presence of PrA, being the binding of the antigen no longer facilitated by the presence of PrA (Figure S2, light blue curve). 

The MWCNT-CSNPs-PrA-BSA platform, in the absence of a D-dimer antibody, was successively tested with a fixed concentration of D-dimer antigen. No significant current signal was obtained, confirming the absence of specific antigen-modified electrode interactions.

#### 3.4.2. EIS Characterization

The Nyquist plots corresponding to each modification step of the immunosensor platform were recorded (Figure 4B) and successfully fitted by the simple Randles circuit (inset Figure 3B). 

A large increase of the semicircle diameter (R_CT_) was observed after the immobilization of PrA through affinity interactions between PrA and CSNPs step (Figure 4B, pink curve), compared to the unmodified MWCNTs-CSNPs electrode (Figure 4B, red curve), due to the electron transfer hindrance caused by the immobilized PrA. Despite this negative effect, the role of PrA is of extreme importance. It is known in the literature that it allows site-orientating and accurate anchoring of a high number of antibody molecules on the electrode surface [37], thanks to the exposure of the Fab active sites to D-dimer epitopes, which results in a strong enhancement of the immunosensor selectivity towards D-dimer antigens. A further increase of the semicircle diameter was observed after the immobilization of the D-dimer antibody and BSA molecules on the electrode surface (Figure 4B, blue curve), attesting to the well-oriented affinity binding of the antibody and the absorption of BSA, which both make the electrode surface more insulating, causing a further electron transfer hindrance.

Lastly, the highest R_CT_ value was obtained after the affinity binding of the D-dimer antigen due to the hindering effect of the Ab–Ag complex towards the redox probe in reaching the electrode surface (Figure 4B, green curve). All EIS data are reported in Table 1 (rows 4–6). Good fitting was confirmed by a χ^2^ value of 0.003. It is possible to note that the R_CT_ value showed a 631%, 714%, and 754% enhancement after PrA, D-dimer antibody-BSA, and D-dimer antigen immobilization, respectively, calculated using the following equation:

ΔR_CT_% = R_CTmod_-R_CTi_/R_CTi_ × 100

where R_CTi_ is the R_CT_ for the MWCNTs-CSNPs-SPE (initial platform), and R_CTmod_ is the charge transfer resistance after each surface modification step. 

### 3.5. Optimization of D-Dimer Electrochemical Platform

To achieve the best D-dimer immunosensor performance, the following parameters were optimized by EIS experiments: (i) antibody concentration; (ii) antibody binding time; (iii) antigen incubation time (Figure S3). By increasing the antibody concentration up to 1.0 μg mL^−1^, the R_CT_ values initially increased (up to 0.1 μg mL^−1^) and then reached a plateau at higher concentrations, indicating the saturation of the electrode surface with increasing antibody concentration (Figure S3, panel A). Therefore, 0.1 μg mL^−1^ was selected as the optimum antibody concentration for the D-dimer immunosensor development. Also, the effect of the antibody binding time was successively tested (Figure S3, panel B). The R_CT_ signal increased from 5 to 20 min. A later time enhancement resulted in no further R_CT_ increase, probably due to the presence of PrA that offered a well-ordered antibody anchorage. As a result, 20 min was selected as the optimal time for antibody immobilization for further experiments.

Finally, the optimum incubation time of the D-dimer antigen and the specific antibody was investigated through varying interaction times, between 5 and 30 min. The maximum response was observed at a minimum incubation time of 15 min (Figure S3, panel C), demonstrating the reaching of the antigen saturation for the optimized antibody concentration. The optimum value of 15 min was therefore used for further experiments. 

### 3.6. Analytical Performance of D-Dimer Immunosensor 

**Figure 5:**
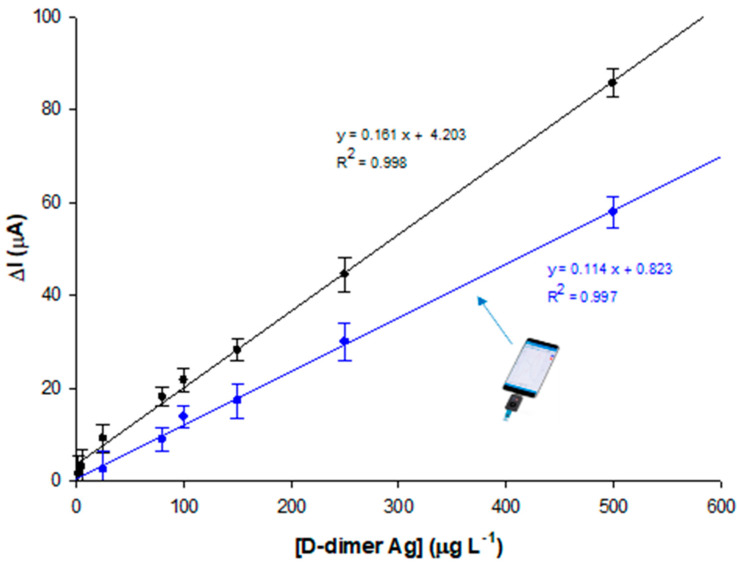
Calibration plot for different D-dimer concentrations (2, 5, 25, 80, 100, 150, 250, and 500 μg L^−1^ in PBS pH 7.4) with Autolab PGSTAT potentiostat (black curve) and Sensit Smart portable potentiostat (blue curve). Experimental conditions: Zobell’s solution; D-dimer antibody = 0.1 μg mL^−1^.

Preliminary experiments were carried out using both DPV and EIS detection techniques, the former showing a faster response time. Therefore, voltammetric detection was utilized for immunosensor development. At increasing D-dimer antigen concentrations, a decrease in the current density of the oxidation redox signal at 150 mV was observed, proving the binding of the antigen to the well-oriented antibody recognition sites, due to PrA. The calibration curve is reported in Figure 5. The immunosensor exhibits a linear relation with D-dimer concentration in the range 2–500 μg L^−1^ with a LOD of 0.6 μg L^−1^, calculated as three times the noise, and a sensitivity of 1.3 μA L μg^−1^ cm^−2^. It is interesting to note that the calibration curve presents a saturation at a concentration of 500 μg L^−1^, which coincides with the clinical cut-off value [36]. Further, the voltammetric immunosensor was used with a portable potentiostat directly connected to a smartphone for POC signal reading, showing a slightly shorter dynamic linear range between 5 and 500 μg L^−1^, a LOD value of 1.6 μg L^−1^, and a slightly lower sensitivity of 0.9 μA L μg^−1^ cm^−2^. 

Table 2 shows a brief overview of the available D-dimer voltammetric sensors. Our results are quite consistent, in terms of linear range, with previous works reported in the literature but exhibit higher LOD values. However, these values are within the clinically relevant range. Therefore, the immunosensor can be used as an alternative for a POC clinical evaluation after a simple dilution step without further sample pretreatment. Moreover, the proposed immunosensor allowed a shorter Ag incubation time equal to 15 min, half the time used for the other immunosensors reported in the literature.

### 3.7. Repeatability, Reproducibility, Stability, and Selectivity of the D-Dimer Immunosensor

The repeatability of the immunosensor was tested by calculating the relative standard deviation (RSD%) value of five (*n* = 5) successive measurements with the same electrode platform at a D-dimer antigen concentration of 5 μg L^−1^ (RSD 1.5%).

As for reproducibility, an RSD of about 2.0% was obtained with five (*n* = 5) different electrode platforms at the same D-dimer antigen concentration, confirming a reasonable reproducibility of our system. 

Then, the stability of the D-dimer platform was determined by storing the electrode at 4 °C for 3 weeks and monitoring it at regular intervals of 3 days. Over the 12th day, the signal remained stable after gradually decreasing with time, reaching about 11% on the 21st day (Figure S4).

**Figure 6:**
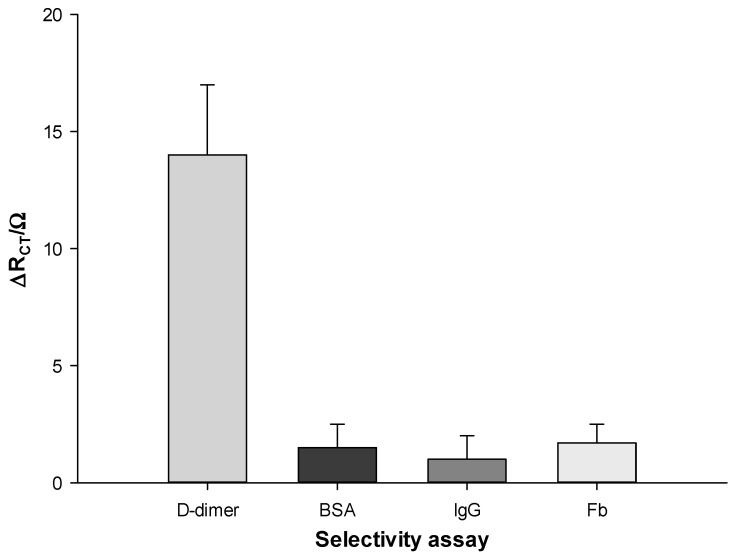
Histogram of selectivity assay for D-dimer antigen (10 μg L^−1^) vs. different interferent proteins (100 μg L^−1^) Zobell’s solution.

Lastly, the selectivity of our platform was studied by comparing the DPV signals of D-dimer to other proteins, such as BSA, human IgG, and fibrinogen (Fb). The DPV signal decreases were registered after 15 min incubation of each protein on the MWCNTS-CSNPs-PrA-Ab-BSA platform. In particular, 10 μg L^−1^ was used for D-dimer protein, and 100 μg L^−1^ for the three interferents tested. These results show the high selectivity of the proposed biosensor, as no significant signals have been registered with the interferents tested, although they have been used at 10 times higher concentrations (Figure 6).

### 3.8. Application in Real Plasma Samples

The proposed portable immunosensor was tested in 5 plasma samples of healthy and COVID-19-infected patients, attested by positive RT-PCR results. The human plasma samples were diluted with PBS pH 7.4 to different concentrations in the range of 10 to 500 μg L^−1^, where the immunosensor shows linearity. Table 3 shows the correlation between the results obtained with the immunosensor and the conventional immunoturbidimetric method. The results show good agreement between the two methods (RSD% values between 1 and 16), indicating that the suggested immunosensor can successfully measure D-dimer levels in human plasma. It must be remarked that high D-dimer levels may not be useful for COVID-19 diagnosis but only for COVID-19 prognosis. A high D-dimer level in a patient who tested negative for COVID-19 is a diagnostic marker for thrombotic diseases, such as DVT, PE, and DIC, whereas a high D-dimer level in a positive patient may represent an important indication to require further exams because of a high risk of a thrombotic event related to COVID-19.

The repeatability and reproducibility of the immunosensor were also tested in each real plasma sample. The results obtained were in good agreement with the values previously reported in standard solutions with RSD% values in the range 2.1–3.6%, 1.9%, and 3.2%, respectively. In Table S1, all replicated data obtained with the plasma sample of patient 4 are reported.

The stability was also checked in real plasma samples, attesting to no significant difference compared to the stability in D-dimer standard solutions. As shown in Figure S4, the current signal remained stable in the first 10 days, showing a gradual small reduction with time, reaching 20% of its initial value after 3 weeks. 

### 3.9. Advantages, Drawbacks, and Future Perspectives of D-Dimer Immunosensor

The most important advantages of the proposed immunosensor are fast response time, simplicity, portability, and stability. The main weakness is the cost of each modified electrode (about Euro 6), which remains too high for a disposable sensor. The future goal is to turn the developed immunosensor into a cost-effective commercial device whose performance is comparable to conventional laboratory testing and which can be used both in hospitals and at home as a diagnostic and prognostic device for thromboembolic events and COVID-19 disease, respectively. Future work should also focus on developing miniaturized portable potentiostats and incorporating portable batteries, thanks to the development of low-cost packaging and manufacturing methods. Further, automating and integrating is another challenging process. By integrating the internet-of-things (IoT), machine learning, cloud computing, data analysis, and artificial intelligence (AI), the proposed nano-immunosensor can lead to a telemedicine scenario.

## 4. Conclusions

A label-free immunosensor was successfully developed to detect D-dimer antigens in clinical range levels. The high performance seems probable due to the CSNPs and MWCNTs synergism that enabled a high conductivity of the nanomodified electrode. Moreover, the strategy to use PrA to immobilize D-dimer antibodies, leading to a better orientation of the antibodies towards epitopes and a consequent immobilization of many biomolecule antigens, resulted in a stable and sensitive platform for D-dimer detection. Several studies have provided robust evidence that D-dimer is a predictive marker of thromboembolic events and of serious complications due to SARS-CoV-2 infection. Therefore, rapid and disposable D-dimer testing is a useful tool for patients arriving at the hospital showing both thromboembolic and COVID-19 symptoms for early triaging to start the correct treatment avoiding complications [40,41,42,43].

The developed sensor showed good linearity within the detection range necessary for diagnosis and prognosis, good sensitivity and reproducibility, and excellent specificity. Furthermore, the disposable immunosensor allowed for the detection of D-dimer antigens in plasma samples without any sample pretreatment, showing satisfactory agreement with the photometric/turbidimetric conventional method. It is suitable for repeated measurements during the day, often required in clinical multi-parameter continuous non-invasive monitoring, as the half-life of D-dimer is approximately 8 h [44]. For these reasons, the developed immunosensor may have great potential as a disposable lab-on-a-chip device for thromboembolic event diagnosis and COVID-19 prognosis. 

## Figures and Tables

**Table 1:** Randles parameters for step-by-step development of D-dimer immunosensor.

MWCNTs-SPE	R_s_ (Ω)	R_CT_ (Ω)	W (μMho s^1/2^)	Q	
				Y0 (μMho)	N
bare	135	324	1.40	45	0.692
CS layer	149	229	292	177	0.616
CSNPs	123	35	647	1160	0.618
CSNPs-PrA	125	256	484	842	0.454
CSNPs-PrA-Ab-BSA	127	285	1314	257	0.541
CSNPs-PrA-Ab-BSA-Ag	136	299	1000	100	0.766

**Table 2:** Voltammetric immunosensors for D-dimer protein detection.

Sensor-Ab	Sample	Ab Binding Time (Min)	Ag Incubation Time (Min)	L.R. (μg L^−1^)	LOD (μg L^−1^)	Ref.
MWCNTs-NTA-Cu	buffer	30	30	0.1–1 × 10^6^	0.1	[29]
Au-ppy-Cu	buffer	30	30	0.01–10	0.01	[38]
Au-ppy-Cu	buffer	30	30	1–500	1	[39]
MWCNTs-CSNPs-PrA	buffer	20	15	2–500	0.6	this work
MWCNTs-CSNPs-PrA (Sensit SMART)	buffer	20	15	5–500	1.6	

List of abbreviations: NTA- nitrile-triacetic-acid; Au- gold electrode; ppy- polypyrrole; Cu- copper complex; MWCNTs- multi-walled carbon nanotubes; CSNPs- chitosan nanoparticles; PrA- Protein A.

**Table 3:** D-dimer determination in human plasma samples using the proposed immunosensor and a conventional immunoturbidimetric reference method.

Sample	D-Dimer Immunosensor (μg L^−1^)	Conventional Method (μg L^−1^)	RSD%	COVID-19 RT-PCR Test
patient 1	539	543	1	-
patient 2	940	1114	16	+
patient 3	170	172	14	-
patient 4	868	950	9	+
patient 5	813	934	13	-

## Data Availability

Not applicable.

## Supplementary Materials

The following supporting information can be downloaded at: https://www.mdpi.com/article/10.3390/bios13010043/s1, Figure S1. Optimization of: (A) D-dimer antibody concentration; (B) antibody binding time; (C) antigen incubation time, Figure S2. Stability assay of the D-dimer immunosensor.
