# Supplementary material for: Disposable Voltammetric Immunosensor for D-Dimer Detection as Early Biomarker of Thromboembolic Disease and of COVID-19 Prognosis
Source: Biosensors (Basel). 2022 Dec 28;13(1):43. doi: 10.3390/bios13010043 (PMC9855840; doi:10.3390/bios13010043)
Supplement: Supplementary file 1 [file biosensors-13-00043-s001.zip › biosensors-2080338-SI.pdf]

biosensors

Supplementary Material

Disposable Voltammetric Immunosensor for D-Dimer Detection as Early Biomarker of Thromboembolic Disease and of COVID-19 Prognosis

Cristina Tortolini [1], Valeria Gigli [1], Antonio Angeloni [1], Luciano Galantini [2], Federico Tasca [3] and Riccarda Antiochia [4,*]

[1] Department of Experimental Medicine, "Sapienza", University of Rome, V.le Regina Elena 324, 00166 Rome, Italy
[2] Department of Chemistry, "Sapienza", University of Rome, P.le Aldo Moro 5, 00185 Rome, Italy
[3] Departamento de Química de los Materiales, Facultad de Química y Biología, Universidad de Santiago de Chile, Casilla 40, Correo 33, Sucursal Matucana, Santiago 9170022, Chile
[4] Department of Chemistry and Drug Technologies, "Sapienza", University of Rome, P.le Aldo Moro 5, 00185 Rome, Italy
* Correspondence: riccarda.antiochia@uniroma1.it

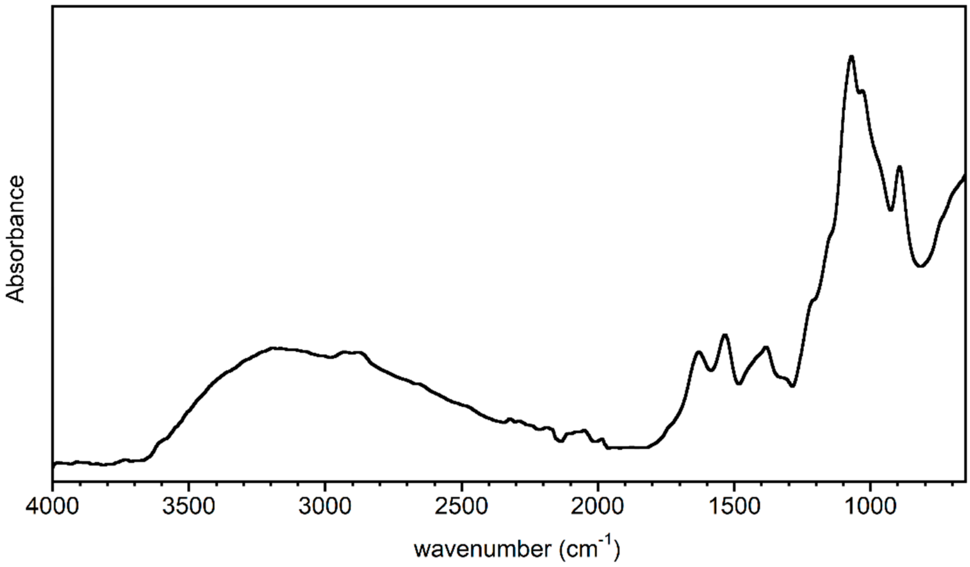

Figure S1. FTIR spectrum of freeze-dried CSNPs.

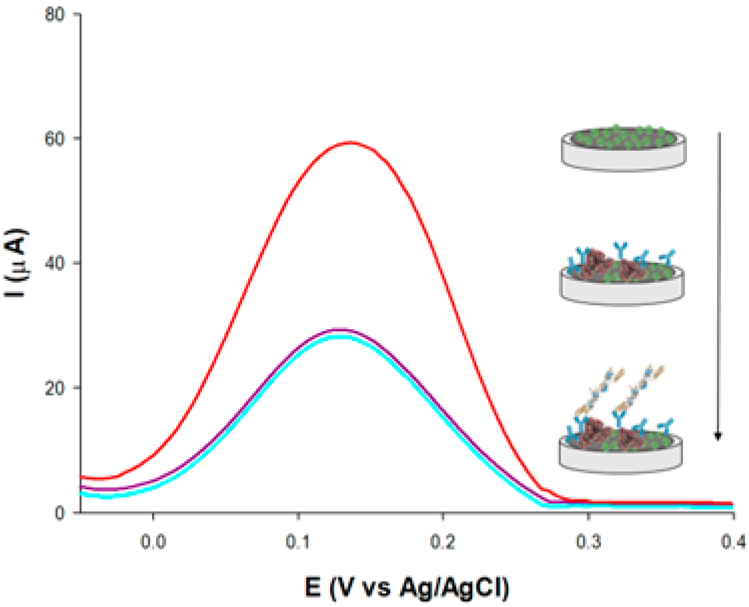

Figure S2. DPVs of: MWCNTs-CSNPs (red), MWCNTs-CSNPs-Ab-BSA (violet), MWCNTs-CSNPs-Ab-BSA-Ag (light blue) SPEs, measured in Zobell's solution.

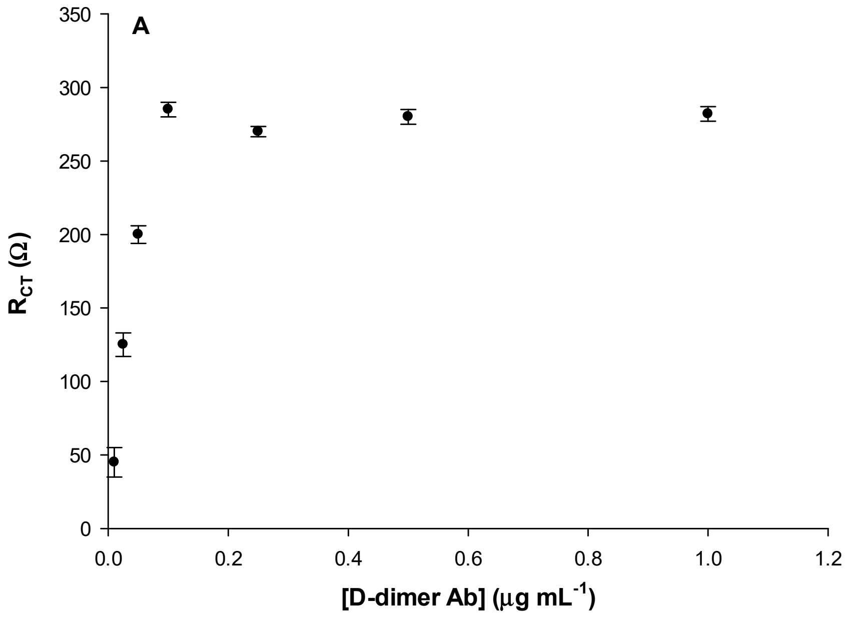

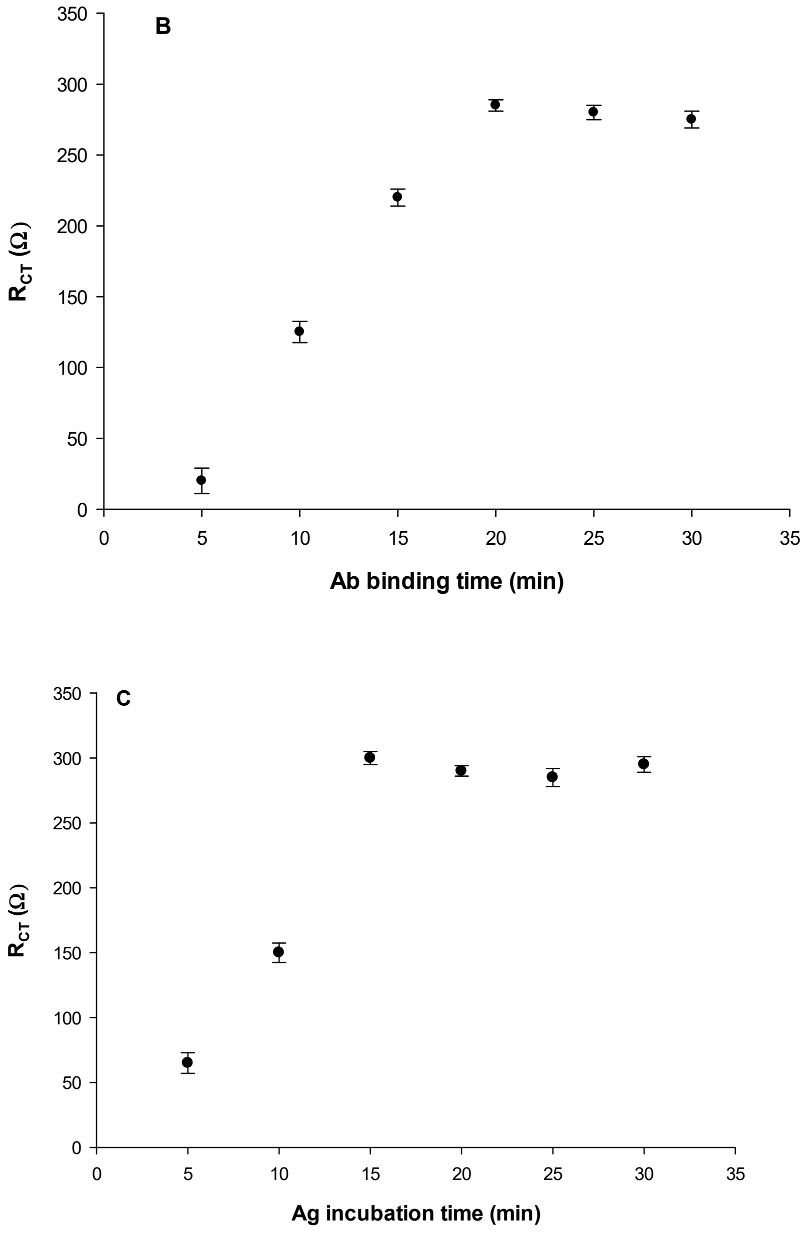

Figure S3. Optimization of: (A) D-dimer antibody concentration; (B) antibody binding time; (C) antigen incubation time. Experimental conditions: Zobell's solution; frequency range: 0.1-105 Hz; AC signal amplitude: 10 mV.

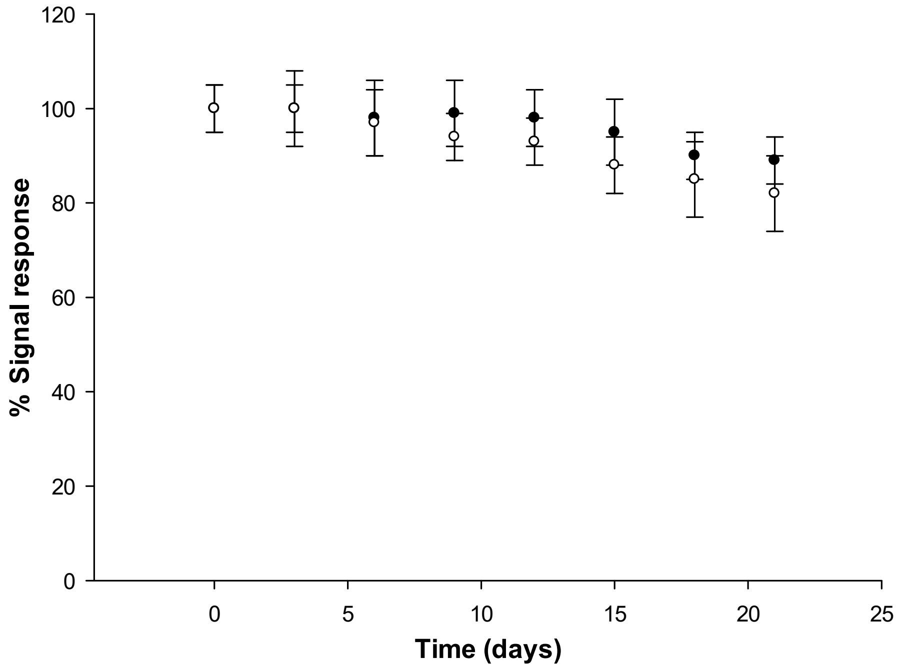

Figure S4. Stability assay of the D-dimer immunosensor for standard solution (●) and for human plasma sample (patient 4, (○). Experimental conditions: Zobell's solution; potential applied: 0.150 V (vs. Ag/AgCl).

Table S1. Riproducibility and repeatibility measurements and parameters for one human plasma sample (patient 4).

**Platform**	**Measure**					**mean**	**SD**	**CV**
	**1**	**2**	**3**	**4**	**5**			
**1**	839	887	856	887	873	868	20.8	2.4
**2**	856	858	894	834	844	857	22.7	2.7
**3**	903	852	843	900	883	876	27.5	3.1
**4**	886	886	851	888	856	873	18.3	2.1
**5**	835	828	899	844	879	857	30.6	3.6
**mean**	864	862	869	871	867			
**SD**	29.7	24.9	25.9	29.5	16.5			
**CV**	3.4	2.9	3.0	3.4	1.9			

* SD: standard deviation; CV: coefficient of variation (SD/mean*100).
